# Proteomics pinpoints alterations in grade I meningiomas of male versus female patients

**DOI:** 10.1038/s41598-020-67113-3

**Published:** 2020-06-25

**Authors:** Janaína M. Silva, Helisa H. Wippel, Marlon D. M. Santos, Denildo C. A. Verissimo, Renata M. Santos, Fábio C. S. Nogueira, Gustavo A. R. Passos, Sergio L. Sprengel, Luis A. B. Borba, Paulo C. Carvalho, Juliana de S. da G. Fischer

**Affiliations:** 1 Laboratory for Structural and Computational Proteomics, Carlos Chagas Institute, Fiocruz, Paraná Curitiba, Brazil; 2 Clinical Hospital of the Federal University of Paraná, Paraná, Brazil; 3 Laboratory of Protein Chemistry, Proteomic Unit, Institute of Chemistry, Federal University of Rio de Janeiro, Rio de Janeiro, Brazil; 4 Hospital Universitário Evangélico Mackenzie, Paraná, Brazil

**Keywords:** Proteins, Proteome, Cancer

## Abstract

Meningiomas are among the most common primary tumors of the central nervous system (CNS) and originate from the arachnoid or meningothelial cells of the meninges. Surgery is the first option of treatment, but depending on the location and invasion patterns, complete removal of the tumor is not always feasible. Reports indicate many differences in meningiomas from male versus female patients; for example, incidence is higher in females, whereas males usually develop the malignant and more aggressive type. With this as motivation, we used shotgun proteomics to compare the proteomic profile of grade I meningioma biopsies of male and female patients. Our results listed several differentially abundant proteins between the two groups; some examples are S100-A4 and proteins involved in RNA splicing events. For males, we identified enriched pathways for cell-matrix organization and for females, pathways related to RNA transporting and processing. We believe our findings contribute to the understanding of the molecular differences between grade I meningiomas of female and male patients.

## Introduction

Meningioma is a high incidence tumor that typically emerges at the arachnoid cap or meningothelial cells of the meninges^1^, commonly from intracranial, intraspinal, or orbital locations, and are usually benign, slow-growing tumors^2^. Resonance imaging and molecular markers are frequently used for preliminary diagnosis; yet, surgical removal of the tumor is necessary for histological diagnostic confirmation and improved life quality^1,2^. When surgery for total removal of the tumor is not feasible, adjuvant radiation may be used^1^.

The World Health Organization (WHO) classifies meningiomas according to their histopathological characteristics, mitotic count, and brain invasion pattern in (i) grade I, also known as benign meningiomas (BMs, about 80% of termed cases); (ii) grade II, or atypical meningiomas (AMs, 17% of termed cases); and (iii) grade III, the malignant meningiomas (MMs, 3% of termed cases)^3^. Although this classification is valid in terms of prognosis, it lacks information about tumor aggressiveness and recurrence rates^2^. Controversially, most recurrent meningiomas correspond to BMs; their metabolic phenotype indicates an aggressive metabolism, resembling that of AM^4^.

Female patients present approximately double the incidence of meningiomas compared to men^1^. Interestingly, the main risk factor for meningiomas is related to hormonal changes as these tumors present hormones receptors (i.e., progesterone and estrogen)^5,6^. Moreover, association between meningiomas and breast cancer, mainly due to similar hormonal signaling and genetic predisposition, has also been reported^5^. The fact that women diagnosed with breast cancer are more likely to develop meningioma may also justify the higher incidence of this tumor in females^7,8^. In general, women usually develop the benign form while males develop the malignant type of meningioma, the aggressive grade III^6^. Besides gender, other risk factors associated with this disease include exposure to ionizing radiation^9^, family history^5^, and porting specific mutations such as the neurofibromatosis type 2 (*NF2*), characterized by a mutation on chromosome 22q12^10^ or in genes involved in the sonic hedgehog and phosphatidylinositol-3 kinase (PI3K)/AKT/mTOR pathways, i.e., *AKT1, PIK3CA*, and *SMARCE1* ^1^.

There are few proteomic studies on meningiomas. Sharma *et al*. characterized the serum proteome of patients with different degrees of meningiomas^11^ and identify differential regulation of important physiological pathways related to coagulation, lipid metabolism and cell growth. The results posed apolipoprotein E and A-I and hemopexin as potential predictors for meningiomas. The proteins vimentin, alpha-2-macroglobulin, apolipoprotein B and A-I, and antithrombin III presented differential abundancy according to the degree of meningioma and may function as predictive markers that complement the histological diagnosis. In another report, Papaioannou *et al*.^12^ evaluated the alteration of meningioma proteins of different degrees; 61 samples were analyzed and a total of 3,042 proteins were identified using a Q-Exactive Plus mass spectrometer (Thermo, San Jose). Dunn *et al*.^13^ also evaluated the proteomic and phosphoproteomic profile of meningiomas (grade I, II, and III) versus healthy meninges and identified 3,888 proteins and 3,074 phosphoproteins.

To date, no proteomic report has addressed the molecular mechanisms related to gender-specific tumorigenesis – much is still hypothesized^14^. With this as motivation, we employed shotgun proteomics to compare the proteomic profile of grade I meningioma biopsies of male versus female patients. Our results showed enriched pathways involved in cell-matrix organization for male samples and RNA splicing and processing for female patients, thus posing as a contribution to the understanding of the molecular differences between genders.

## Results

### Exploratory data analysis

We performed a Clustergram (hierarchical clustering + heatmap), Principal Component Analysis (PCA), and a t-distribution Distributed Stochastic Neighbor Embedding (t-SNE) analyses on our dataset. This is accomplished by encoding data from the technical replicates of each biological replicate into a vector whose dimensions hold normalized quantitation values of the identified proteins. PCA and t-SNE are commonly used for dimensional reduction to provide a visual interpretation of the dataset. PCA projects vectors to a reduced set of orthogonal axis linked to maximum variance. t-SNE uses the t-distribution to group objects in the higher dimensional space and then relies on Kullback–Leibler divergence minimize the projections to a corresponding distribution at a lower dimension; t-SNE is know for producing more effective visualizations. The Clustergram was achieved using PatternLab’s Clustergram module with the Feature Stringency Selection parameter set to 0.95^15^. The PCA and t-SNE were generated using DiagnoProt 2.0; in brief this software clusters spectra and performs downstream analyses on these vectorized clusters and thus is search-engine unbiased^16^. These results are all provided in Supplementary File 1.

### Quality assessment of technical replicate reproducibility

The study includes six biological replicates for each condition; each biological replicate served for generating two technical replicates. We then used RawVegetable (Freely available at: http://www.patternlabforproteomics.org/rawvegetable/) to certify that the reproducibility of all 12 technical replicate pairs achieve a reproducibility RawVegetable k-score <0.1. The scatterplots of all analyses are provided as Supplementary File 2.

### Unique proteins identified in female and male meningiomas

We performed our proteomic analysis following the PatternLab for proteomics protocol^15^. A summary of our identification numbers is reported in Table 1, and the complete report on Supplementary file 3.

**Table 1:** Summary of the identification results.

Group	Spectra	Peptides	Proteins	Proteins (Max. Pars)
Female	146,790	27,395	4,046	3,379
Male	148,494	28,054	3,913	3,233

Groups: Data from 6 female and 6 male grade I meningiomas. The Spectra, Peptides, Proteins, and Proteins (MaxPars) reflect the number of identifications. MaxPars stands for Maximum Parsimony (i.e., the minimum number of proteins that explains all the identified peptides).

**Figure 1:**
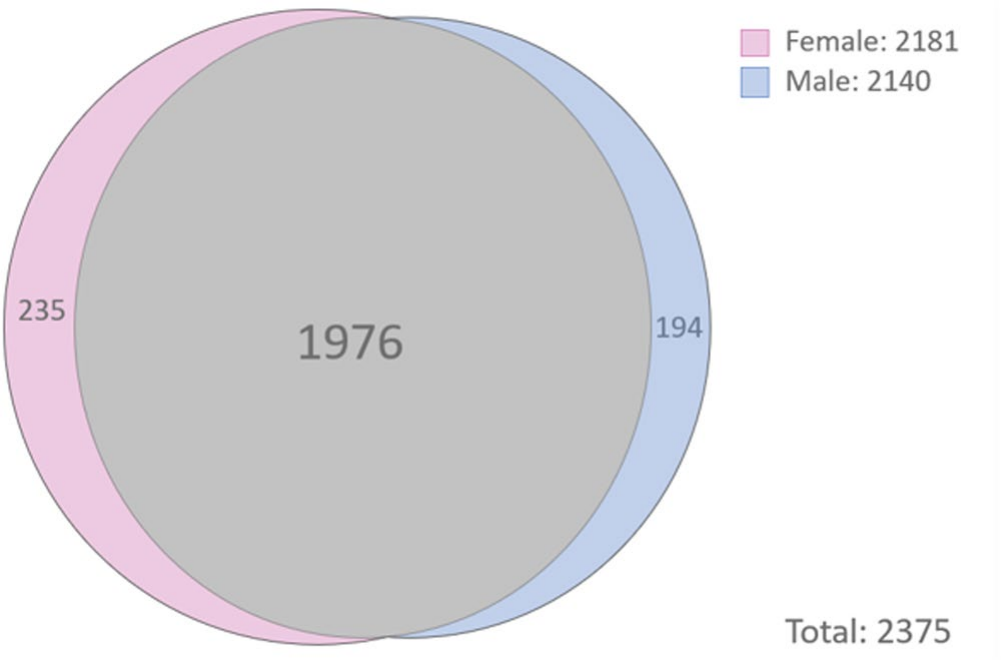
Venn diagram of the meningioma samples from the patients included in the study. Female group with 235 exclusive proteins. Male group with 194 exclusive proteins. A total of 1,946 proteins are common to female and male groups.

We used PatternLab’s “Venn Diagram” to pinpoint proteins identified in two or more biological replicates of each biological condition; the results indicated 235 and 194 exclusive to the female and male groups, respectively (Fig. 1; complete information in Supplementary file 3). Table 2 shortlists proteins distinct to each gender that is related to cancer, cell growth or cell cycle, as per DAVID platform (complete information in Supplementary file 4).

**Table 2:** Uniquely identified proteins in female and male groups.

Unique Male	Description	Unique Female	Description
ALOX5AP	Arachidonate 5-lipoxygenase-activating protein	DDX3×	DEAD-Box Helicase 3 ×-Linked
C2	Complement C2	EIF4G2	Eukaryotic Translation Initiation Factor 4 Gamma 2
C6	Complement C6	MMP14	Matrix metalloproteinase-14
C8G	Complement C8 Gamma Chain	NCBP1	Nuclear Cap Binding Protein Subunit 1
DCN	Decorin	GAB1	GRB2-associated-binding protein 1
FAS	Tumor necrosis factor receptor superfamily member 6	LARP1	La-related protein 1
HLA-DPA1	HLA class II histocompatibility antigen, DP alpha 1 chain	CDK5RAP3	CDK5 regulatory subunit-associated protein 3
IFI16	Gamma-interferon-inducible protein 16	MRE11	Double-strand break repair protein
IL18	Interleukin-18	PDS5B	Sister chromatid cohesion protein PDS5 homolog B
ISG15	Ubiquitin-like protein	SRC	Proto-oncogene tyrosine-protein kinase Src
ITGAV	Integrin alpha-V	ZPR1	Zinc finger protein
ITGB3	Integrin beta-3	SCRIB	Protein scribble homolog
LAMC1	Laminin subunit gamma-1	TACC1	Transforming acidic coiled-coil-containing protein 1
LBP	Lipopolysaccharide-binding protein	GPS1	COP9 signalosome complex subunit 1
LCN2	Neutrophil gelatinase-associated lipocalin	CUL4B	Cullin-4B
LTBP1	Latent-transforming growth factor beta-binding protein 1	MCTS 1	Malignant T-cell-amplified sequence 1

Proteins identified uniquely in male and female meningiomas related to cell cycle, cell growth or cancer according to analysis by the DAVID platform.

### Differentially abundant proteins identified in female and male meningiomas

**Figure 2:**
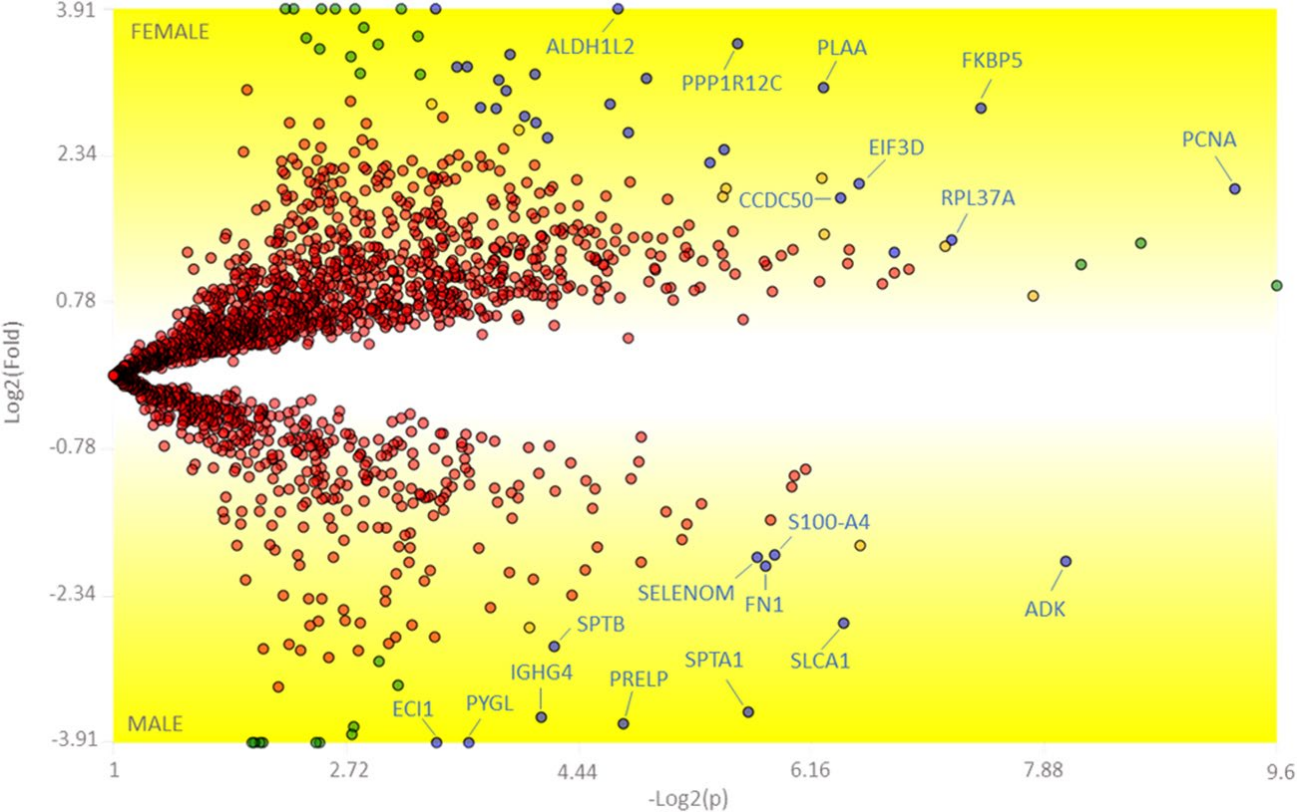
Differently abundant proteins identified in the female and male groups. The y- and x- axis are related to the fold change and p-value. Red dots are proteins that do not satisfy our fold-change cutoff and the established False Discovery Rate (FDR). Green dots are proteins whose abundancy fold change satisfy the criteria but not the FDR. The 37 blue dots represent proteins that satisfy both criteria.

We used PatternLab’s TFold analysis to assess the differences in protein abundances identified between the groups evaluated (Supplementary file 3). Figure 2 shows the graph corresponding to protein distribution under the two conditions studied. A total of 37 proteins were identified as differently abundant (blue dots), described in Table 3; of those, 11 were upregulated in the male group and 26 in the female group.

**Table 3:** Differently abundant proteins identified in female and male patient groups.

Protein ID	Fold change	Protein description
ECI1	−18.88	Enoyl-CoA delta isomerase 1, mitochondrial
PYGL	−16.33	Glycogen phosphorylase, liver form
PRELP	−13.04	Prolargin
IGHG4	−12.42	Immunoglobulin heavy constant gamma 4
SPTA1	−11.96	Spectrin alpha chain, erythrocytic 1
SPTB	−7.37	Spectrin beta chain, erythrocytic
SLC2A1	−6.2	Solute carrier family 2, facilitated glucose transporter member 1
FN1	−4.07	Fibronectin
ADK	−3.92	Adenosine kinase
SELENOM	−3.81	Selenoprotein
S100A4	−3.74	Protein S100-A4
TXNDC17	2.48	Thioredoxin domain-containing protein 17
RPL37A	2.72	60 S ribosomal protein L37a
CCDC50	3.71	Coiled-coil domain-containing protein 50
PCNA	3.96	Proliferating cell nuclear antigen
EIF3D	4.12	Eukaryotic translation initiation factor 3 subunit D
SNX3	4.81	Sorting nexin-3
NEFL	5.3	Neurofilament light polypeptide
SF3B1	5.78	Splicing factor 3B subunit 1
RAB4B	6.01	Ras-related protein Rab-4B
SPART	6.46	Spartin
TJP2	6.77	Tight junction protein ZO-2
EPB41L1	7.18	Band 4.1-like protein 1
FKBP5	7.2	Peptidyl-prolyl cis-trans isomerase FKBP5
DUT	7.23	Deoxyuridine 5’-triphosphate nucleotidohydrolase, mitochondrial
AP1G1	7.41	AP-1 complex subunit gamma-1
SORBS1	8.19	Sorbin and SH3 domain-containing protein 1
PLAA	8.38	Phospholipase A-2-activating protein
PRKAR2A	8.86	cAMP-dependent protein kinase type II-alpha regulatory subunit
LZTFL1	8.97	Leucine zipper transcription factor-like protein 1
CPNE1	9.25	Copine-1
HSPH1	9.76	Heat shock protein 105 kDa
ANXA3	9.77	Annexin A3
RAP1GDS1	10.7	Rap1 GTPase-GDP dissociation stimulator 1
PPP1R12C	11.59	Protein phosphatase 1 regulatory subunit 12C
FAU	17.12	40 S ribosomal protein S30
ALDH1L2	29.46	Mitochondrial 10-formyltetrahydrofolate dehydrogenase

Protein ID: Swiss-Prot protein identifier. Fold change: ratio between female and male protein NIAF values; negative values indicate greater abundance in the male group compared to the female group. Protein description: according to the Swiss-Prot database. All proteins satisfies a *q*-value <0.1.

### Enriched pathways in female and male meningiomas

The proteins identified to only one gender or presenting differential abundancy were selected to search for enriched pathways using the Reactome software. Tables 4 and 5 list the enriched pathways together with their corresponding identified proteins for female and male meningioma samples, respectively. For the female group, most are related to RNA processing and transport, whereas for the male group, we highlight the extracellular matrix (ECM)-related pathways. The complete information of the Reactome analysis is available in Supplementary file 5.

**Table 4:** Pathways enriched in female meningioma.

Pathway name	Identified proteins
Processing of Capped Intron-Containing Pre-mRNA	BCAS2, CPSF3, CSTF2, DDX42, DDX46, NCBP1, NUP205, NUP93, NUP98, PRPF31, PRPF6, SF3B1, SF3B4, SF3B5, U2AF2
Transport of Mature mRNAs Derived from Intron less transcripts	CPSF3, NCBP1, NUP205, NUP93, NUP98
Signaling by EGFR	ARHGEF7, GAB1, PLCG1, PTPN12, SRC, STAM2
Transport of the SLBP Dependent Mature mRNA	NCBP1, NUP205, NUP93, NUP98
Vpr-mediated nuclear import of PICs	NUP205, NUP93, NUP98, PSIP1
tRNA processing in the nucleus	CSTF2, NUP205, NUP93, NUP98, XPOT

The five major pathways enriched in female meningioma were selected by importing all the exclusive and differentially abundant proteins into Reactome.

**Table 5:** Pathways enriched in male meningioma.

Pathway name	Identified proteins
Extracellular matrix organization	AGRN, COL12A1, COL14A1, COL6A2, COLGALT1, CTSS, DCN, ELANE, FBLN2, FBLN5, FN1, HSPG2, ITGAM, ITGAV, ITGB3, LAMB2, LAMC1, LTBP1, MFAP5, NID2, PECAM1
Molecules associated with elastic fibres	FBLN2, FBLN5, FN1, ITGAV, ITGB3, LTBP1, MFAP5
ECM proteoglycans	AGRN, COL6A2, DCN, FN1, HSPG2, ITGAV, ITGB3, LAMB2, LAMC1
Elastic fibre formation	FBLN2, FBLN5, FN1, ITGAV, ITGB3, LTBP1, MFAP5
Non-integrin membrane-ECM interactions	AGRN, FN1, HSPG2, ITGAV, ITGB3, LAMB2, LAMC1
Integrin cell surface interactions	AGRN, COL6A2, FN1, HSPG2, ITGAM, ITGAV, ITGB3, PECAM1

The five major pathways enriched in male meningioma were selected by importing all the exclusive and differentially abundant proteins into Reactome.

## Discussion

### Protein alterations in male meningioma

#### Apolipoprotein L1

The human apolipoprotein L1 (APOL1) was uniquely identified in male grade I meningiomas (Supplementaty file 3). Apolipoproteins are glycoproteins that bind lipids to form and transport lipoproteins, acting in the homeostasis of lipid and lipoprotein metabolism in the liver^17^. APOL1 is a minor component of high density lipoprotein (HDL), but despite its main role in lipid transport and metabolism, recent studies have shown a role of APOL1 in apoptosis, innate immunity, and autophagy, all processes related to cancer, due to its similarity to Bcl-2 family proteins^18^.

Specifically, in meningiomas a great variety of apolipoproteins have been identified with differential abundance in a study that used serum quantitative proteomics to compare WHO grades I-III meningiomas^11^. Some examples are the APOE and A-I, considered potential predictors for meningiomas. APOB and A-I were differentially abundant in malignant grades of meningioma, posing as a potential biomarker for the disease. Also, apolipoproteins A-I, A-II, A-IV, B-100, C-II and E were altered in atypical and anaplastic meningiomas. In reference to other studies, APOE and APOA-I were also found with differential abundance in WHO grade I-III meningiomas^19,20^

#### Extracellular matrix (ECM) organization

Tumor cells show changes in cell-cell and cell-ECM adhesion processes, thus contributing to cancer progression from loss of contact with their original tissues^21^. Our data indicate that in male meningioma there are mostly changes in cell-ECM adhesion, while in females, there is an enrichment for RNA processing-related processes (Tables 5 and 6).

**Table 6:** Details of patients included in this study: ID, age, gender, and diagnosis.

ID	Age (years)	Gender	Diagnostic
1	67	Female	Fibroblastic Meningioma (Grade I)
2	54	Female	Meningothelial Meningioma (Grade I)
3	58	Female	Parasagital Meningioma (Grade I)
4	66	Female	Unspecified meningioma (Grade I)
5	74	Female	Meningothelial Meningioma (Grade I)
6	83	Male	Meningothelial Meningioma (Grade I)
7	83	Male	Meningothelial Meningioma (Grade I)
8	76	Male	Meningothelial Meningioma (Grade I)
9	63	Male	Transitional Meningioma (Grade I)
10	59	Male	Unspecified meningioma (Grade I)
11	45	Male	Angiomatous Meningioma (Grade I)
12	88	Female	Meningothelial Meningioma (Grade I)

ECM is responsible for cell-cell communication, adhesion and cell proliferation and is highly modified by remodeling and degradation processes, with its regulation or dysregulation has direct effects on cell differentiation and adhesion^22^. The composition of ECM varies according to the needs of the tissue, which is remodeled according to biochemical signals^23^. The proteins decorin, integrin alpha-M, fibronectin, microfibrillar-associated protein 5, laminin subunit gamma-1, among others, participate in the organization of ECM and were identified exclusively or upregulated in male meningioma (Supplementary file 3). Integrins are responsible for anchoring cells to the ECM, and fibronectins connect integrins to other ECM proteins^24^. Our study identified integrin beta 3 precursor (ITGB3), alpha V integrin (ITGAV) and alpha M integrin (ITGAM) only in male meningioma samples (Table 2); these proteins are associated with meningiomas tumorigenesis 7,8. Integrins function as transmembrane receptors mediating ECM adhesion and other cellular processes, such as cell migration and angiogenesis 7. Reports indicate the ECM constitution to be altered in tumor cells, and an increase in fibronectin secretion to be noted; here, we identified this upregulation pattern in the male group^24^. In a recent study, proteins involved in ECM formation and were found differentially abundant in grade I meningiomas^12^.

Tumor cells are suggested to infiltrate the ECM and promote biochemical changes that increase metastatic spread^25^, and perhaps these events are related to the greater aggressiveness of male meningiomas.

#### Neutrophil degranulation

Proteins involved in neutrophil degranulation, such as ARSA, GCA, FUCA1, PRTN3, ELANE, MNDA, and LCN2, were identified uniquely or with differential abundance in male meningiomas (Supplementary file 3). Neutrophils are the first defense line cell population to reach the site of inflammation and are capable of binding to tumor cells, providing greater angiogenesis, cell matrix remodeling and tumor progression^26^. They secrete three types of granulocytes that modulate cell function, called primary, secondary and tertiary^27^. Primary factors mainly secrete myeloperoxidases, proteolytic proteins, and bactericides; secondary and tertiary factors secrete proteins that interact and degrade the cellular matrix, such as metalloproteinase-9 (MMP-9)^26,27^.

Papaioannou and collaborators reported the enrichment of the neutrophil degranulation pathway in aggressive meningiomas, based on the time of recurrence^12^. Templeton and collaborators showed that a neutrophil/lymphocyte ratio of more than 4 in the peripheral blood of patients with different types and stages of cancer is correlated with a poor prognosis and survival^28^. However, in the tumor microenvironment, it is not known if the presence of neutrophils is related to the worsening of prognosis. Shen and collaborators evaluated the solid tumor neutrophil population of 3,946 patients with different cancers and concluded that increased intratumoral neutrophil levels are associated with decreased patient survival^29^.

#### CCN family member 3 (NOV)

CCN family member 3 (NOV) was identified uniquely in male meningiomas (Supplementary file 3). CCN family proteins are involved in cell adhesion, proliferation, and differentiation^30^. Under normal biological conditions, NOV is related to neuronal and muscular differentiation^31^. However, given pathological stimuli, it is suggested that the protein is involved in tumorigenic events^31^. Thibout and collaborators showed that NOV levels are significantly higher in malignant adrenocortical tumors when compared to benign tumors, revealing the participation of NOV in the aggressiveness and worse prognosis of the disease^32^. The role of NOV in tumorigenesis was investigated by Dankner and collaborators; they analyzed samples from 1,500 patients with primary prostate cancer and concluded that NOV protein abundance correlates with bone metastasis events^33^. These data corroborate the findings of Chen and collaborators, that demonstrated that NOV silencing decreases tumor growth^34^. Positively regulated NOV is related to a poor prognosis of cervical cancer^35^ and metastatic primary musculoskeletal tumors^36^.

However, the effects of NOV are indicated to be beneficial in cases of glioblastomas, as *in vitro* assays have shown that the protein has antiproliferative effects and prevents the S/G2 transition in the cell cycle^37^. Fukunaga-Kalabis and collaborators. indicated that NOV prevented melanoma cell invasion and that reduced NOV levels may facilitate the invasive character of melanoma cells^38^. In addition, the protein had antiproliferative effects in gliomas^39^ and Wilms tumors^40,41^. These findings suggest NOV may decrease cell proliferation and increase apoptotic events. NOV interacts with different proteins and participates in different cellular events, depending on the type of cell in which it is located, and this may be the reason for its dual role in tumorigenesis^41^.

NOV interacts with the Notch-1 transmembrane receptor and thus promote downstream effects on the Notch signaling pathway^42^ that is linked to embryonic cell development, coordinating cell differentiation, cell proliferation, and apoptosis. Liao and collaborators showed that Notch-1 silencing inhibits cell growth and promotes apoptosis in HT29 cells, a model colorectal carcinoma cell culture^43^. However, data from Sin and collaborators showed that NOV reduces cell growth by regulating actin cytoskeleton reorganization and increasing intercellular adhesion in breast cancer cells^44^. These studies show different mechanisms of NOV protein actions, revealing a close relationship with tumor development.

#### S100-A4

The S100 protein family is composed of proteins that play key roles in regulating cell events, such as cell cycle progression, and are described at multiple stages of tumorigenesis^45,46^. One of the CCN3 interaction partners is the calcium-binding protein S100-A4^47^, which in our study was identified with a higher abundancy in male meningioma (Table 4). S100-A4 is involved in cell cycle control, angiogenesis, motility, and cell adhesion, and therefore, related to tumor progression and metastasis^48^. Albeit S100-A4 having a role in tumorigenesis and metastasis^49^, this protein is also found in normal human cells, such as macrophages, fibroblasts, granulocytes, and T lymphocytes^49^. The interaction between S100-A4 and p53 promotes p53 degradation^50^, an important tumor suppressor. Loss of protein function prevents the cell cycle from progressing moving on to the next phase, which may result in the development of tumors^51–53^, such as brain tumors^54^, breast^55^, colon^56^ and lung^57^ carcinomas. S100-A4 poses as a strong biomarker candidate to indicate early tumor detection and also offers possible evidence of metastatic events of these tissues, being identified in the breast^58^, brain^59^, and liver^60^ cancer metastasis.

Metastasis events related to the action of S100-A4 linked to its intra and extracellular functions^61^. In the extracellular context, the protein may be related to cytokine recruitment^62^ and inflammation, increased secretion of growth factors in the tumor environment, and increased angiogenesis^63,64^. In addition, the tumor cells themselves can secrete S100A4 and stimulate angiogenesis, favoring nutrient and oxygen exchange, as well as the disposal of metabolites and carbon dioxide^49^. In the intracellular context, S100-A4 covalently binds to actins, non-muscular myosin IIA, and tropomyosin and together alter cell migration and adhesion^48^. The reduction in S100A4 levels is reported to correlate with the decrease in epithelial-mesenchymal transition (EMT) events^65^. EMT is a process in which an epithelial cell assumes a mesenchymal phenotype, and then presents greater migratory capacity and invasiveness, and also presents apoptosis resistance. Non-muscular myosin IIA protein regulates EMT events, and may be related to increased EMT events, thus potentially contributing to metastatic events^65,66^.

The alterations in the proteomic profile of male meningiomas when compared to the female group may be related to the higher cancer aggressiveness and poor prognosis of male patients.

### Protein alterations in female meningioma

#### RNA splicing and transport

We identified RNA splicing and transport-related proteins as uniquely identified or differentially abundant in the female group (Supplementary file 3). RNA splicing is a very important mechanism for increasing proteome diversity, and its proper control is required to maintain cellular processes; several studies suggest that aberrant splicing of some genes may be related to cancer^67^. We have identified differentially abundant protein interactions related to RNA processing, such as DDX42, BCAS2, DDX3×, PRPF6, PRPF31 (Supplementary file 3). In a study using quantitative proteomics to compare WHO grades I, II and III meningiomas, proteins involved in RNA splicing/processing were found differentially abundant in malignant grades II-III meningiomas, when compared to benign grade I^12^.

Reports indicate a close relationship between the occurrence of meningiomas and stimulation by hormones, especially estrogens^68^. Estrogen receptors (ERs) are associated with cases of meningiomas and breast cancer, but there are more studies in cases of breast tumors^69^. In general, ER-alpha is described as a transcription factor that increases cell growth and proliferation, while ER-beta performs antiproliferative functions^70^. Dago and collaborators performed RNA sequencing to evaluate the difference in cell transcripts expressing only ER-alpha or ER-alpha and ER-beta in response to estradiol hormone, which is a natural estrogen^71^; the results indicated that both forms induced RNA splicing in response to estradiol, and that ER-beta positive cells exhibited about twice as many mRNA splicing events as receptor negative cells.

As aforementioned, meningiomas of female patients have more progesterone receptors (PRs) than those of male patients^72^. Other results indicate that the presence of PR is higher in benign tumors and that PR status is inversely related to mitotic intensity and degree of meningiomas^73^. The same was reported for breast cancer, where decreased PR is related to a worse prognosis. Loss of PR can be related to several factors, such as PR promoter hypermethylation and PR pre-mRNA alternative splicing events, which can generate receptor variants with different domains, and thus modify how the cells respond to progesterone, contributing to the growth and abnormal proliferation of these cells^73^.

#### The proliferative cell nuclear antigen (PCNA)

The protein called proliferative cell nuclear antigen (PCNA) has multiple functions, including DNA repair and replication, chromatin remodeling and cell cycle regulation^74^. In our study, the protein was identified as differentially abundant in female compared to male meningioma. Studies have found that ER-alpha is associated with PCNA, and Norton-Schultz and collaborators showed that PCNA helps maintain the basal expression of estrogen-responsive genes^75^. In breast cancer, ER-alpha has been shown to affecting the cell cycle by suppressing p53 / p21 activity, and by increasing the levels of PCNA and KI-67 antigen (Ki-67)^76^. It has also been found that stimulation of ER-alpha by 17-β-estradiol increases MCF-7 cell proliferation by increasing PCNA and Ki-67 levels^76^. This information provides important clues as to how stimulation by hormones can influence the development of meningioma, especially the female, which is more frequent.

## Conclusions

Our study compared the proteomes of biopsies derived from meningiomas of male and female patients. Most of the proteins were identified in both genders (82%); those uniquely identified in female meningiomas pointed to enriched pathways related to RNA splicing and transport-related pathways; and have been described as enriched for breast cancer and related to hormone exposure. The enriched pathways in the male group were related to extracellular matrix organization which are linked to cancer aggressiveness and metastasis. We recall that proteins uniquely identified in one condition does not mean a complete absence in the other one but only that they were not identified by our approach; regardless, this is still suggestive of differential abundancy^77^. We also point out that exclusively identified proteins to a single biological condition does not mean that they are fully absent in the other; their absence could be due to the stochastic nature of data-dependent acquisition or with an abundancy lower than the detection limits of our approach.

## Material and Methods

### Materials

Qubit Protein Assay Kit (Cat. no Q33212) and RapiGest SF acid-labile surfactant (Cat. no 186001861) were purchased from Invitrogen (Carlsbad, CA) and Waters Corp. (Milford, MA), respectively. Sequence grade modified trypsin (V511A) was purchased from Promega. All other laboratory reagents were acquired from Sigma-Aldrich (St. Louis, MO), unless specified otherwise.

### Patients

This study was approved by the ethics committee of Oswaldo Cruz Foundation and the Federal University of Clinical Hospital of Curitiba under the numbers 63056316.8.0000.5248 and 63056316.8.3001.0096, respectively. A written informed consent was acquired from each patient. As such, all methods were carried out in accordance with relevant guidelines and regulations for this manuscript. The tumor fragments were collected by neurosurgeons belonging to the clinical staff of the Clinical Hospital of the Federal University of Paraná. The collected samples were stored in sterile 15 mL capped tubes, which were transported on dry ice, following all necessary biosecurity standards for such procedure. All collected material was aliquoted using sterile material and then stored at -80 °C. All patients were diagnosed with type I meningioma, as shown in the Table 6.

### Sample preparation

The twelve tissue samples of grade I meningiomas were pulverized in liquid nitrogen, as previously described^78^. Then, protein extraction was made in a solution of 0.1% of RapiGest (w/v) in 50 mM triethylammonium bicarbonate (TEAB). Subsequently, the extracted proteins were centrifuged at 18,000 × *g* at 4 °C, for 15 minutes and supernatant was collected. The protein content was quantified by a fluorimetric assay using the Qubit 2.0 platform, according to the manufacturer’s instructions. Next, 180 µg of total protein from each sample was reduced with 10 mM of dithiothreitol (DTT) at 60 °C for 30 minutes. Then, all samples were cooled to room temperature and incubated in the dark with 25 mM of iodoacetamide (IAA) for 30 minutes. The samples were subsequently digested for 20 hours with high sequence grade modified trypsin at a 1:50 (Enzyme/Substrate) ratio at 37 °C. Following digestion, all reactions were acidified with 10% (v/v) trifluoroacetic acid (0.5% v/v final concentration) to stop proteolysis and precipitate RapiGest. The samples were centrifuged for 15 minutes at 18,000 × *g* at 20 °C to remove insoluble materials. Then, peptides were desalted with a C18 spin column, according to the manufacturer’s instructions (Harvard Apparatus).

### Mass spectrometry analysis

We used a nanoLC Easy1000 coupled online with a Q-Exactive plus mass spectrometer to generate two proteomic profiles of each biological replicate using the same materials and methods as we previously described^78^.

### Reproducibility assessment

The study consisted of 12 biological samples; six from male and six from female grade I meningioma biopsies. For each biological sample, two technical replicates were generated, producing a total of 24 Q-Exactive Plus runs. All technical replicates were assessed for reproducibility; those achieving RawVegetable’s reproducibility k-score below 0.1 were repeated. PatternLab’s bioinformatic analysis merges the information from the technical replicates to reduce under sampling.

### Peptide spectrum matching (PSM)

Our bioinformatic analysis was guided by the steps described in the PatternLab for proteomics protocol^15^; the software version we used was PaternLab for proteomics 4.1.1.17 that is freely available at http://www.patternlabforproteomics.org. The *H. Sapiens* Swiss-Prot database^79^ was downloaded on February 19th, 2019; a reversed version of each sequence plus those from 127 common mass spectrometry contaminants was included. The search considered semi-tryptic and fully-tryptic peptide candidates, allowing a maximum of 2 lost cleavage sites. Oxidation of methionine and carbamidomethylation of cysteine were considered as variable and fixed modifications, respectively.

### Validation of PSMs

The Search Engine Processor (SEPro), built into PatternLab, was used for converging to a list of identifications with less than 1% of false discovery rate (FDR) at the protein level, as previously described^80^. Briefly, the identifications were grouped by charge state (2 + and ≥3 +), and then by tryptic status, resulting in four distinct subgroups. For each group, the XCorr, DeltaCN, DeltaPPM, and Peaks Matched values were used to generate a Bayesian discriminator. The identifications were sorted in non-decreasing order according to the discriminator score. A cutoff score was established to accept a false-discovery rate (FDR) of 1% at the peptide level based on the number of labeled decoys. This procedure was independently performed on each data subset, resulting in an FDR that was independent of charge state or tryptic status. Additionally, a minimum sequence length of six amino-acid residues was required. Results were post-processed to only accept PSMs with less than 10 ppm from the global identification average. One-peptide identifications (i.e., proteins identified with only one mass spectrum) with the peptide having an XCorr of less than 2 were discarded. This last filter led to FDRs, now at the protein level, to be lower than 1% for all search results.

### Relative quantitation of proteins

Quantitation was performed according to PatternLab’s Normalized Ion Abundance Factors (NIAF) as a relative quantitation strategy. We recall that NIAF is the equivalent to NSAF^81^, but applied to extracted ion chromatogram (XIC)^82^. The PatternLab TFold module^83^ was used to pinpoint differentially abundant proteins between the female and male groups. The proteins log fold change was estimated by obtaining the log of the averaged corresponding peptide folds. Our differential proteomic comparison only considered proteins identified with two or more unique peptides (i.e., peptides that map to a single sequence in the database), a *q*-value ≤ 0.1 and an absolute peptide fold change cutoff > 3. Only proteins present in at least two technical replicates (from six biological replicates for each gender) were considered for the TFold analysis. In summary, the procedure briefly described in our bioinformatics protocol^15^. Finally, we used the Reactome^84^, DAVID^85^ tools to help interpret the data.

## Data Availability

The mass spectrometry proteomics data have been deposited to the ProteomeXchange Consortium via the PRIDE^86^ partner repository with the dataset identifier PXD015979.

## Supplemenatry information

Supplemenatry information1.
Supplemenatry information2.
Supplemenatry information3.
Supplemenatry information4.
Supplemenatry information5.
