# Supplementary material for: Proteomics pinpoints alterations in grade I meningiomas of male versus female patients
Source: Sci Rep. 2020 Jun 25;10:10335. doi: 10.1038/s41598-020-67113-3 (PMC7316823; doi:10.1038/s41598-020-67113-3)
Supplement: Supplementary file 1 — Supplemenatry information1. [file 41598_2020_67113_MOESM1_ESM.docx]

**Supplementary Material.** **Clustergram, PCA, and t-SNE analysis**

**Proteomics pinpoint alterations in grade I meningiomas of male versus female patients**

Janaína M. Silva^1&^, Helisa H. Wippel^1&^, Marlon D. M. Santos^1^, Denildo C. A. Verissimo^1,2^, Renata M. Santos^3^, Fábio C. S. Nogueira^3^, Gustavo A. R. Passos^4^, Sergio L. Sprengel^2^, Luis A. B. Borba^2,4^, Paulo C. Carvalho^1*^, Juliana de S. da G. Fischer^1*^

**Figure 1** provides a PCA, t-SNE, and a Radial Basis Function mapping of the t-SNE results. **Figure 2** provides a Clustergram as per PatternLab for proteomics^1^ with the Feature Stringency Selection parameter set to 0.95. and is followed inline with Table I that provides detailed information on each protein considered when generating the Clustergram.

**Figure 1-A**


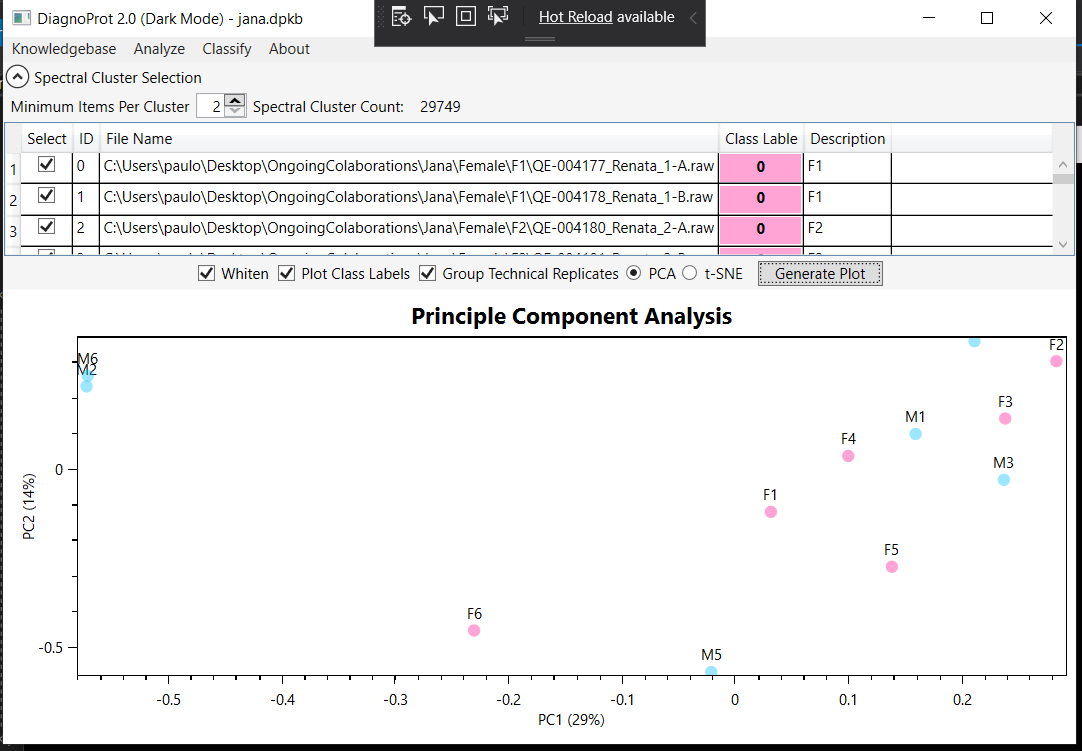


**Figure 1-B**


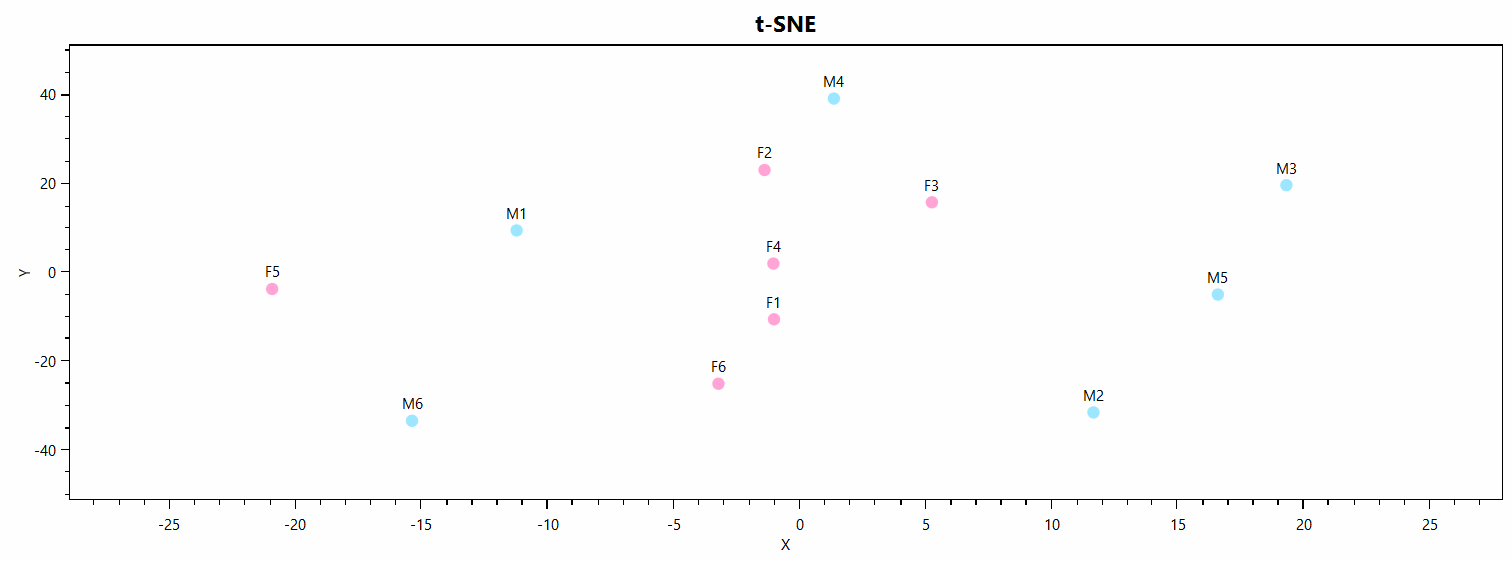


**Figure 1-C**


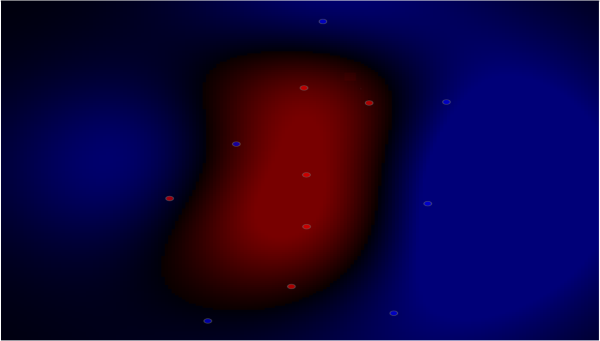


**Figure 1.** Panel A demonstrates a PCA plot of the male and female proteomic samples. Female and Male samples are represented in Red and Blue, respectively. Panel B provides the t-SNE plot of the same data and Panel C a Radial Basis Function interpretation of the t-SNE results.

**
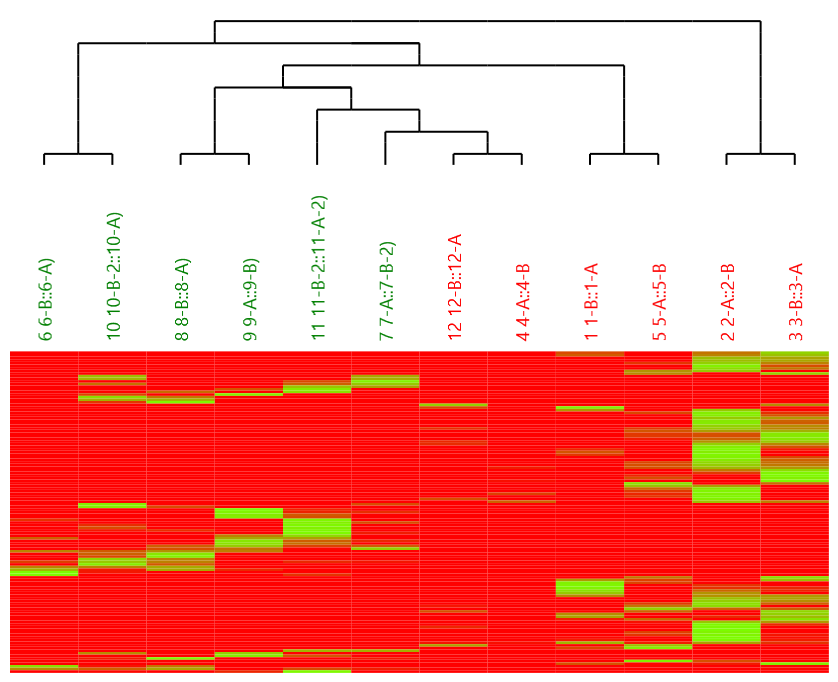
**

**Figure 2.** Clustergram as per PatternLab for proteomics. Columns labeled in green and red correspond to proteomic analysis derived from male and female patients, respectively. The 123 rows correspond to normalized protein quantitation values; green represents a higher abundancy. Table I provides details on the proteins from each row and is ordered accordingly.

**Table I**

| Protein ID | Quant Values | Description |
| --- | --- | --- |
| P51784 | 2.64467295381039E-06, 0, 4.15337802795691E-06, 7.49328055505656E-06, 0, 0, 0, 0, 0, 0, 0, 0 | Ubiquitin carboxyl-terminal hydrolase 11 |
| Q9NTZ6 | 4.11853516604633E-06, 0, 5.91670149949665E-06, 8.71874316090526E-06, 0, 0, 0, 0, 0, 0, 0, 0 | RNA-binding protein 12 |
| O43592 | 0, 0, 6.45840895619126E-06, 5.5607751487284E-06, 0, 0, 0, 0, 0, 0, 0, 0 | Exportin-T |
| Q7Z478 | 0, 0, 5.33096387096766E-06, 4.67678399861365E-06, 0, 0, 0, 0, 0, 0, 0, 0 | ATP-dependent RNA helicase DHX29 |
| Q08378 | 0, 0, 5.1243712117742E-06, 3.24559193076472E-06, 0, 1.31068112635697E-06, 0, 0, 0, 0, 0, 0 | Golgin subfamily A member 3 |
| Q9UKV8 | 0, 0, 5.52939338464871E-06, 1.74072299719995E-06, 0, 0, 0, 0, 0, 0, 0, 0 | Protein argonaute-2 |
| Q96RL7 | 0, 0, 3.92706993243522E-06, 2.16907777813763E-06, 0, 0, 0, 0, 0, 0, 0, 0 | Vacuolar protein sorting-associated protein 13A |
| Q15477 | 0, 0, 3.91039447653237E-06, 0, 0, 2.88179033585251E-06, 0, 0, 0, 0, 0, 0 | Helicase SKI2W |
| O15020 | 0, 0, 0, 1.53899760131575E-06, 0, 1.0263615258376E-06, 0, 0, 0, 0, 0, 0 | Spectrin beta chain, non-erythrocytic 2 |
| P20929 | 0, 0, 0, 0, 0, 0, 4.66541960082871E-07, 0, 0, 4.06041224761632E-07, 0, 0 | Nebulin |
| Q5JSH3 | 0, 0, 0, 0, 0, 0, 1.57246930131879E-06, 0, 0, 1.50603566089699E-06, 0, 0 | WD repeat-containing protein 44 |
| P16284 | 0, 0, 0, 0, 0, 0, 0, 9.63288333872855E-07, 0, 3.20791396434164E-06, 0, 0 | Platelet endothelial cell adhesion molecule |
| P04066 | 0, 0, 0, 0, 0, 0, 2.61501836844207E-06, 2.12857382161375E-06, 0, 4.52581706431663E-06, 0, 0 | Tissue alpha-L-fucosidase |
| P35612 | 0, 0, 0, 0, 0, 0, 0, 4.98539194580735E-06, 0, 3.95083395220107E-06, 0, 0 | Beta-adducin |
| P21980 | 0, 0, 0, 0, 0, 0, 0, 5.59674277583363E-06, 0, 0, 0, 2.03059370621505E-06 | Protein-glutamine gamma-glutamyltransferase 2 |
| Q14766 | 0, 0, 0, 0, 0, 0, 0, 2.18850258104828E-06, 0, 0, 1.03199173842822E-06, 0 | Latent-transforming growth factor beta-binding protein 1 |
| P55268 | 0, 0, 0, 0, 0, 0, 5.4113491983752E-07, 0, 0, 0, 0, 1.95662796784479E-06 | Laminin subunit beta-2 |
| Q0VD83 | 0, 0, 0, 0, 0, 0, 3.09144117524009E-06, 0, 0, 0, 1.68410062559791E-06, 0 | Apolipoprotein B receptor |
| Q8WWM7 | 0, 0, 0, 0, 0, 0, 3.0768797401335E-06, 0, 0, 0, 3.13997767787521E-06, 0 | Ataxin-2-like protein |
| Q3KQU3 | 0, 0, 0, 0, 0, 0, 1.96834005875683E-06, 0, 0, 0, 9.0171212214058E-06, 0 | MAP7 domain-containing protein 1 |
| Q01433 | 0, 4.61810372044741E-06, 0, 3.04473602799027E-06, 0, 0, 0, 0, 0, 0, 0, 0 | AMP deaminase 2 |
| P34896 | 6.86856520810562E-06, 1.97593046701873E-06, 0, 0, 0, 0, 0, 0, 0, 0, 0, 0 | Serine hydroxymethyltransferase, cytosolic |
| P33240 | 6.71045929586303E-06, 0, 8.82964509180277E-06, 0, 0, 0, 0, 0, 0, 0, 0, 0 | Cleavage stimulation factor subunit 2 |
| Q9H2M9 | 0, 0, 8.85186090810238E-06, 1.7018398474026E-06, 0, 2.60739367206214E-06, 0, 0, 0, 0, 0, 0 | Rab3 GTPase-activating protein non-catalytic subunit |
| Q5JTZ9 | 0, 0, 9.69898910010652E-06, 3.38600397936792E-06, 0, 0, 0, 0, 0, 0, 0, 0 | Alanine--tRNA ligase, mitochondrial |
| Q29RF7 | 0, 0, 8.23520687187435E-06, 6.23205134073764E-06, 0, 0, 0, 0, 0, 0, 0, 0 | Sister chromatid cohesion protein PDS5 homolog A |
| O94906 | 0, 0, 1.29664020306636E-05, 5.52538276682629E-06, 0, 0, 0, 0, 0, 0, 0, 0 | Pre-mRNA-processing factor 6 |
| P51610 | 0, 0, 1.45595078790721E-05, 5.66779439363099E-06, 0, 0, 0, 0, 0, 0, 0, 0 | Host cell factor 1 |
| Q96RP9 | 0, 0, 1.40737099092676E-05, 9.42661405722347E-06, 0, 0, 0, 0, 0, 0, 0, 0 | Elongation factor G, mitochondrial |
| Q14161 | 0, 3.49176260352908E-06, 1.45275993236361E-05, 9.15740455712661E-06, 0, 4.01289712051352E-06, 0, 0, 0, 0, 0, 0 | ARF GTPase-activating protein GIT2 |
| Q6EMK4 | 0, 0, 1.21877851706541E-05, 1.36761529542753E-05, 0, 6.57307996454764E-06, 0, 0, 0, 0, 0, 0 | Vasorin |
| Q9UID3 | 0, 0, 6.9198432288548E-06, 2.02984491573895E-05, 0, 4.83047883568065E-06, 0, 0, 0, 0, 0, 0 | Vacuolar protein sorting-associated protein 51 homolog |
| P51114 | 0, 0, 7.09530124361702E-06, 2.17034051075722E-05, 0, 5.32124907953261E-06, 0, 0, 0, 0, 0, 0 | Fragile X mental retardation syndrome-related protein 1 |
| Q7L014 | 0, 0, 9.03499927550232E-06, 2.16256580787737E-05, 0, 2.54730064441312E-06, 0, 0, 0, 0, 0, 0 | Probable ATP-dependent RNA helicase DDX46 |
| Q9GZT9 | 0, 4.45900214944522E-06, 1.55703148470757E-05, 2.21355718791676E-05, 0, 0, 0, 0, 0, 0, 0, 0 | Egl nine homolog 1 |
| P57764 | 0, 4.60376055014225E-06, 2.13684988790644E-05, 1.38137364528233E-05, 0, 0, 0, 0, 0, 0, 0, 0 | Gasdermin-D |
| Q86SQ0 | 0, 0, 1.97717800291962E-05, 6.67986699275537E-06, 0, 0, 0, 0, 0, 0, 0, 0 | Pleckstrin homology-like domain family B member 2 |
| O60610 | 3.86179046501846E-06, 0, 2.5457485898239E-05, 2.74876339949082E-06, 0, 0, 0, 0, 0, 0, 0, 0 | Protein diaphanous homolog 1 |
| Q13976 | 1.0029868125647E-05, 0, 2.86952370526138E-05, 0, 0, 0, 0, 0, 0, 0, 0, 0 | cGMP-dependent protein kinase 1 |
| Q86UU1 | 7.43706807651951E-06, 3.55742297061685E-07, 3.3558034179422E-05, 0, 0, 0, 0, 0, 0, 0, 0, 0 | Pleckstrin homology-like domain family B member 1 |
| Q8TED9 | 0, 0, 3.14695177743705E-05, 9.29447686516528E-06, 0, 0, 0, 0, 0, 0, 0, 0 | Actin filament-associated protein 1-like 1 |
| Q9Y6A4 | 0, 0, 3.23448596540883E-05, 1.32466952426703E-05, 0, 0, 0, 0, 0, 0, 0, 0 | Cilia- and flagella-associated protein 20 |
| Q9NPQ8 | 0, 0, 3.11641598587077E-05, 1.55792966883818E-05, 0, 9.99012864052773E-06, 0, 0, 0, 0, 0, 0 | Synembryn-A |
| P49585 | 0, 0, 2.89064026904873E-05, 1.67700218756544E-05, 0, 1.13097855659212E-05, 0, 0, 0, 0, 0, 0 | Choline-phosphate cytidylyltransferase A |
| Q96RQ3 | 0, 0, 2.39687225637587E-05, 1.97053542903926E-05, 3.61152988021039E-06, 1.01996757043728E-05, 0, 0, 0, 0, 0, 0 | Methylcrotonoyl-CoA carboxylase subunit alpha, mitochondrial |
| Q9Y2T2 | 0, 0, 1.78750552712455E-05, 3.30240023891145E-05, 0, 0, 0, 0, 0, 0, 0, 0 | AP-3 complex subunit mu-1 |
| Q13464 | 0, 0, 3.35948325877486E-06, 1.49384166635256E-05, 0, 0, 0, 0, 0, 0, 0, 0 | Rho-associated protein kinase 1 |
| Q8IUD2 | 0, 0, 0, 1.66034532519386E-05, 0, 2.41676932845595E-06, 0, 0, 0, 0, 0, 0 | ELKS/Rab6-interacting/CAST family member 1 |
| Q5W0V3 | 0, 0, 0, 9.3760430860999E-06, 0, 3.08861358496293E-06, 0, 0, 0, 0, 0, 0 | Protein FAM160B1 |
| Q9P2D3 | 0, 0, 0, 9.78118200089727E-06, 5.51885328424387E-07, 0, 0, 0, 0, 0, 0, 0 | HEAT repeat-containing protein 5B |
| Q8NHH9 | 0, 0, 0, 4.33270610406493E-06, 0, 8.71580978421685E-06, 0, 0, 0, 0, 0, 0 | Atlastin-2 |
| O60826 | 0, 0, 1.10064328182068E-05, 0, 0, 1.36095019276838E-05, 0, 0, 0, 0, 0, 0 | Coiled-coil domain-containing protein 22 |
| P19784 | 0, 0, 1.97252681055089E-05, 0, 0, 9.08848825678665E-06, 0, 0, 0, 0, 0, 0 | Casein kinase II subunit alpha' |
| Q9H7D7 | 0, 0, 2.37605262110033E-05, 0, 0, 5.60889000456624E-06, 0, 0, 0, 0, 0, 0 | WD repeat-containing protein 26 |
| Q9NSK0 | 0, 0, 1.76181894826687E-05, 0, 4.82772000209397E-06, 2.79343358692516E-06, 0, 0, 0, 0, 0, 0 | Kinesin light chain 4 |
| Q96KP1 | 0, 0, 1.22772112790352E-05, 0, 0, 2.65393677553081E-06, 0, 0, 0, 0, 0, 0 | Exocyst complex component 2 |
| P12931 | 0, 4.25687459908713E-06, 1.30319027985373E-05, 0, 0, 0, 0, 0, 0, 0, 0, 0 | Proto-oncogene tyrosine-protein kinase Src |
| Q9H993 | 0, 0, 1.25132807299979E-05, 8.25940190740483E-06, 6.30389728579292E-06, 0, 0, 0, 0, 0, 0, 0 | Damage-control phosphatase ARMT1 |
| Reverse_Q9Y485 | 0, 0, 0, 0, 0, 0, 1.04610297799228E-05, 0, 0, 3.41270099483953E-06, 0, 0 | |
| Q8TD55 | 0, 0, 0, 0, 0, 0, 1.35758865576911E-05, 0, 0, 0, 4.28519607678096E-06, 0 | Pleckstrin homology domain-containing family O member 2 |
| P00748 | 0, 0, 0, 0, 0, 0, 0, 1.69027812558155E-06, 0, 0, 0, 1.15635730506551E-05 | Coagulation factor XII |
| Q14112 | 0, 0, 0, 0, 0, 0, 0, 1.8868281575155E-06, 0, 2.61430953138926E-06, 0, 1.40409194540405E-05 | Nidogen-2 |
| Q96SM3 | 0, 0, 0, 0, 0, 0, 0, 8.56522636716334E-06, 0, 0, 0, 2.2649605364231E-05 | Probable carboxypeptidase X1 |
| P18428 | 0, 0, 0, 0, 0, 0, 0, 7.92774158483531E-06, 0, 0, 0, 2.37513888275932E-05 | Lipopolysaccharide-binding protein |
| Q86UX2 | 0, 0, 0, 0, 0, 0, 0, 1.24378633278759E-05, 2.44690651156165E-06, 0, 0, 0 | Inter-alpha-trypsin inhibitor heavy chain H5 |
| P49913 | 0, 0, 0, 0, 0, 0, 0, 1.20807990421033E-05, 0, 3.98214317684882E-06, 0, 0 | Cathelicidin antimicrobial peptide |
| Q8NBJ5 | 0, 0, 0, 0, 0, 0, 3.52369723323211E-06, 1.88774524390356E-05, 0, 0, 0, 0 | Procollagen galactosyltransferase 1 |
| Q99538 | 0, 0, 0, 0, 0, 0, 8.1167282122944E-06, 2.60984496176078E-05, 0, 0, 0, 0 | Legumain |
| Q9H3G5 | 0, 0, 0, 0, 0, 0, 3.69131968272347E-06, 3.87866502311502E-05, 0, 0, 7.40731619183333E-06, 0 | Probable serine carboxypeptidase CPVL |
| P28676 | 0, 0, 0, 0, 0, 0, 0, 5.06535802518497E-05, 0, 5.83899032403994E-06, 0, 0 | Grancalcin |
| P98095 | 0, 0, 0, 0, 0, 0, 0, 3.90670623024901E-05, 0, 0, 0, 2.3078346042297E-05 | Fibulin-2 |
| A0A0C4DH68 | 0, 0, 0, 0, 0, 0, 0, 4.6924179766179E-05, 2.9238376605727E-05, 0, 0, 4.95049663574181E-05 | Immunoglobulin kappa variable 2-24 |
| P05546 | 0, 0, 0, 0, 0, 0, 0, 3.49513212244885E-05, 0, 1.51681019634629E-05, 0, 8.61678326692131E-05 | Heparin cofactor 2 |
| Q9NRN5 | 0, 0, 0, 0, 0, 0, 0, 5.58963444125511E-05, 0, 0, 0, 0.000114289603876632 | Olfactomedin-like protein 3 |
| P01602 | 0, 0, 0, 0, 0, 0, 0, 2.31338345953323E-05, 0, 3.31536838892959E-05, 3.361642834455E-05, 7.16877479306284E-05 | Immunoglobulin kappa variable 1-5 |
| P01877 | 0, 0, 0, 0, 0, 0, 3.85230315995545E-06, 2.01329629259047E-06, 0, 5.94684579693682E-05, 2.27537438836419E-05, 3.14553356286882E-05 | Immunoglobulin heavy constant alpha 2 |
| P17812 | 0, 0, 0, 0, 0, 0, 1.20122949597955E-05, 0, 1.74902945102962E-05, 0, 1.8964856158633E-05, 1.69332266940259E-05 | CTP synthase 1 |
| Q9BY43 | 0, 0, 0, 0, 0, 0, 9.91314521617373E-06, 0, 0, 0, 2.3812160097666E-05, 0 | Charged multivesicular body protein 4a |
| Q9BXV9 | 0, 0, 0, 0, 0, 0, 8.64114998542349E-06, 0, 0, 0, 2.97607573621641E-05, 0 | EKC/KEOPS complex subunit GON7 |
| Q8N5M1 | 0, 0, 0, 0, 0, 0, 3.08108975798575E-05, 0, 0, 0, 1.84116000160726E-05, 0 | ATP synthase mitochondrial F1 complex assembly factor 2 |
| Q86Y82 | 0, 0, 0, 0, 0, 0, 5.21108181934729E-05, 1.04382563695992E-05, 1.65666672876517E-05, 5.60732663577834E-06, 3.29888344820473E-05, 0 | Syntaxin-12 |
| Q9BPY8 | 0, 0, 0, 0, 0, 0, 0.000110698012606677, 0, 0, 0, 4.1429229091992E-05, 0 | Homeodomain-only protein |
| P05114 | 0, 0, 0, 0, 0, 0, 7.95542861597688E-05, 0, 8.45375087873295E-05, 0, 0.000107310892524431, 0 | Non-histone chromosomal protein HMG-14 |
| P48745 | 0, 0, 0, 0, 0, 0, 2.2982489180491E-05, 0, 0.000175560199015988, 0, 0.000162510928515432, 3.94444921550095E-05 | CCN family member 3 |
| P10109 | 0, 0, 0, 0, 0, 0, 1.39320834712278E-05, 0, 0.000150845634022704, 0, 0, 0 | Adrenodoxin, mitochondrial |
| Q9Y2R0 | 0, 0, 0, 0, 0, 0, 0, 1.65850071649998E-05, 7.75354117509651E-05, 0, 0, 0 | Cytochrome c oxidase assembly factor 3 homolog, mitochondrial |
| Q9BSD7 | 0, 0, 0, 2.33878680356516E-05, 0, 1.23723562098944E-05, 0, 0, 0, 0, 0, 0 | Cancer-related nucleoside-triphosphatase |
| Q8WWY3 | 1.65511410144244E-05, 0, 0, 2.08969846568611E-05, 0, 6.92697036719347E-06, 0, 0, 0, 0, 0, 0 | U4/U6 small nuclear ribonucleoprotein Prp31 |
| O75352 | 3.07611440080353E-05, 0, 0, 0, 0, 8.75837134293636E-06, 0, 0, 0, 0, 0, 0 | Mannose-P-dolichol utilization defect 1 protein |
| P42226 | 2.9543155617851E-05, 0, 6.47560158641344E-06, 0, 0, 0, 0, 0, 0, 0, 0, 0 | Signal transducer and activator of transcription 6 |
| Q9H1B7 | 4.27142812079869E-05, 0, 8.30784069258294E-06, 6.8190860895881E-06, 0, 0, 0, 0, 0, 0, 0, 0 | Probable E3 ubiquitin-protein ligase IRF2BPL |
| Q9H553 | 3.97473643792119E-05, 0, 1.76186849896728E-05, 6.41655576314173E-06, 0, 0, 0, 0, 0, 0, 0, 0 | Alpha-1,3/1,6-mannosyltransferase ALG2 |
| Q02539 | 3.22497279682051E-05, 0, 2.32219768578431E-05, 0, 0, 0, 0, 0, 0, 0, 0, 0 | Histone H1.1 |
| Q9HAU0 | 2.73339348067181E-05, 0, 2.87825486206074E-05, 3.02014918092744E-05, 0, 0, 0, 0, 0, 0, 0, 0 | Pleckstrin homology domain-containing family A member 5 |
| Q9H2G2 | 0, 0, 4.09803048589415E-05, 3.51112509631554E-05, 0, 0, 0, 0, 0, 0, 0, 0 | STE20-like serine/threonine-protein kinase |
| Q9P1Z2 | 0, 0, 5.08119373394946E-05, 3.51817701478511E-05, 0, 0, 0, 0, 0, 0, 0, 0 | Calcium-binding and coiled-coil domain-containing protein 1 |
| P43897 | 0, 0, 5.17864977999563E-05, 2.2030769803124E-05, 0, 1.42002736965833E-05, 0, 0, 0, 0, 0, 0 | Elongation factor Ts, mitochondrial |
| Q8TE77 | 0, 4.3009889203647E-06, 4.49868318139718E-05, 0, 0, 2.45852036465812E-05, 0, 0, 0, 0, 0, 0 | Protein phosphatase Slingshot homolog 3 |
| Q9NUV7 | 0, 0, 1.06902745822218E-05, 1.04061853942245E-05, 0, 2.32649110170473E-05, 0, 0, 0, 0, 0, 0 | Serine palmitoyltransferase 3 |
| Q8TBF2 | 0, 1.35005470661435E-05, 0, 3.52990520310864E-05, 0, 1.6643138065452E-05, 0, 0, 0, 0, 0, 0 | Prostamide/prostaglandin F synthase |
| Q8NFW8 | 3.57380477205171E-05, 0, 0, 5.37929288542912E-05, 0, 0, 0, 0, 0, 0, 0, 0 | N-acylneuraminate cytidylyltransferase |
| O75934 | 5.18115444249753E-05, 0, 1.25834904627126E-05, 5.98057469265369E-05, 0, 0, 0, 0, 0, 0, 0, 0 | Pre-mRNA-splicing factor SPF27 |
| Q14194 | 1.15615292414448E-05, 0, 0, 7.9725387327676E-05, 0, 3.65474566634949E-05, 0, 0, 0, 0, 0, 0 | Dihydropyrimidinase-related protein 1 |
| Q96L92 | 0, 0, 7.1154690165807E-05, 5.3786297757239E-05, 0, 5.20534997743138E-06, 0, 0, 0, 0, 0, 0 | Sorting nexin-27 |
| Q96JB5 | 0, 0, 8.05554584042439E-05, 1.28063104153824E-05, 0, 0, 0, 0, 0, 0, 0, 0 | CDK5 regulatory subunit-associated protein 3 |
| Q9Y2Y0 | 0, 1.51831864485468E-05, 9.36717816172091E-05, 0, 0, 0, 0, 0, 0, 0, 0, 0 | ADP-ribosylation factor-like protein 2-binding protein |
| Q05209 | 0, 1.29170404374658E-06, 0.000107431764226989, 0, 0, 0, 0, 0, 0, 0, 0, 0 | Tyrosine-protein phosphatase non-receptor type 12 |
| Q9BQ69 | 0, 0, 8.54109528567646E-05, 0, 0, 2.25022693168704E-05, 0, 0, 0, 0, 0, 0 | ADP-ribose glycohydrolase MACROD1 |
| Q13825 | 0, 0, 0.000147695558589068, 1.28191875632384E-05, 0, 3.15091802301123E-05, 0, 0, 0, 0, 0, 0 | Methylglutaconyl-CoA hydratase, mitochondrial |
| Q9NTI5 | 5.45502810905552E-06, 0, 0.000221355727138109, 1.01294757227866E-05, 0, 0, 0, 0, 0, 0, 0, 0 | Sister chromatid cohesion protein PDS5 homolog B |
| Q8WVY7 | 2.4983818996687E-05, 0, 0.000229938603254209, 2.44044113445548E-05, 0, 0, 0, 0, 0, 0, 0, 0 | Ubiquitin-like domain-containing CTD phosphatase 1 |
| Q53EL6 | 6.95543612264761E-05, 7.60729013162626E-06, 6.07972612533409E-05, 1.92762562996313E-05, 5.37028021742242E-06, 1.06300605936965E-05, 0, 0, 0, 0, 0, 0 | Programmed cell death protein 4 |
| P02008 | 0, 4.31830193813191E-05, 0, 0, 0, 5.19941867820788E-05, 0, 0, 0, 0, 0, 0 | Hemoglobin subunit zeta |
| Q8WWI5 | 2.81800675249098E-05, 0, 0, 0, 0, 0.000100972516610307, 0, 0, 0, 0, 0, 0 | Choline transporter-like protein 1 |
| Q9NZD4 | 0, 0, 0, 0, 0, 0, 0, 6.71520655384736E-05, 0, 6.50373656942559E-05, 0, 0 | Alpha-hemoglobin-stabilizing protein |
| Q7LG56 | 0, 0, 0, 0, 0, 0, 6.71095268515529E-05, 0, 0, 0, 0, 9.95593440291923E-05 | Ribonucleoside-diphosphate reductase subunit M2 B |
| Q08431 | 0, 0, 0, 0, 0, 0, 0, 7.84514356860115E-05, 0, 0, 0, 0.000211733560981316 | Lactadherin |
| P49789 | 0, 0, 0, 0, 0, 0, 1.95034132931739E-05, 0, 0, 1.96723116156613E-05, 0.000182858419303866, 0 | Bis(5'-adenosyl)-triphosphatase |
| O43272 | 0, 0, 8.85146525773457E-05, 0, 0, 0.000263304820793122, 0, 0, 0, 0, 0, 0 | Proline dehydrogenase 1, mitochondrial |
| P11172 | 9.65448107514222E-05, 0, 2.90979344145429E-05, 0.000278570934648347, 0, 2.57603025674589E-06, 0, 0, 0, 0, 0, 0 | Uridine 5'-monophosphate synthase |
| L0R6Q1 | 0, 0, 0, 0, 0, 0, 3.28211936268169E-05, 0, 0.000369311717170301, 0, 0.00018907598017299, 0 | SLC35A4 upstream open reading frame protein |
| P49207 | 0, 0, 0, 0, 0, 0, 0.000213586726531014, 0.000162135923273479, 0.000312454003930739, 6.77512625186895E-05, 0.000362344865639949, 0 | 60S ribosomal protein L34 |
| Q05707 | 0, 0, 0, 0, 0, 0, 1.53311799798015E-05, 0.000486850407536118, 0, 0, 0, 7.24255463087097E-05 | Collagen alpha-1(XIV) chain |
