# Supplementary material for: Proteomics pinpoints alterations in grade I meningiomas of male versus female patients
Source: Sci Rep. 2020 Jun 25;10:10335. doi: 10.1038/s41598-020-67113-3 (PMC7316823; doi:10.1038/s41598-020-67113-3)

**Supplementary Material.** **Clustergram, PCA, and t-SNE analysis**

**Proteomics pinpoint alterations in grade I meningiomas of male versus female patients**

Janaína M. Silva^1&^, Helisa H. Wippel^1&^, Marlon D. M. Santos^1^, Denildo C. A. Verissimo^1,2^, Renata M. Santos^3^, Fábio C. S. Nogueira^3^, Gustavo A. R. Passos^4^, Sergio L. Sprengel^2^, Luis A. B. Borba^2,4^, Paulo C. Carvalho^1*^, Juliana de S. da G. Fischer^1*^

This document displays correlation plots generated from technical replicate proteomic runs. Each dot corresponds to an identified protein and is mapped according to the sum of the normalized Extracted Ion Chromatogram (XIC) values from each replicate.


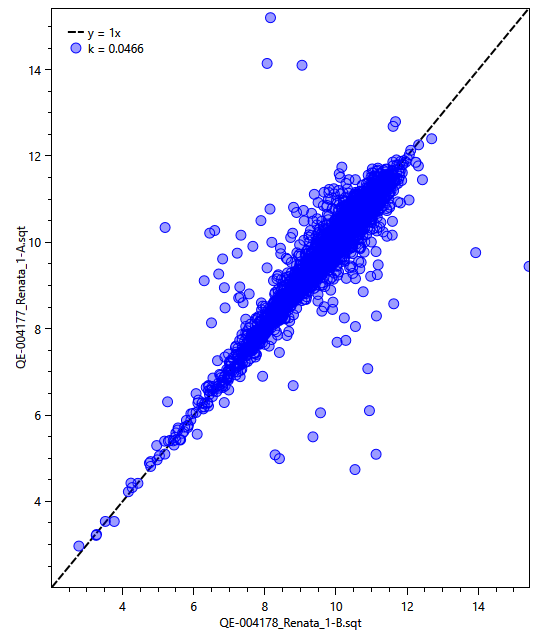

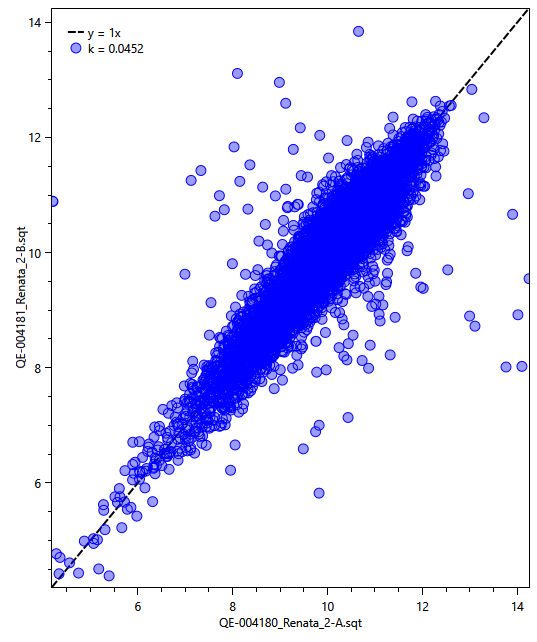

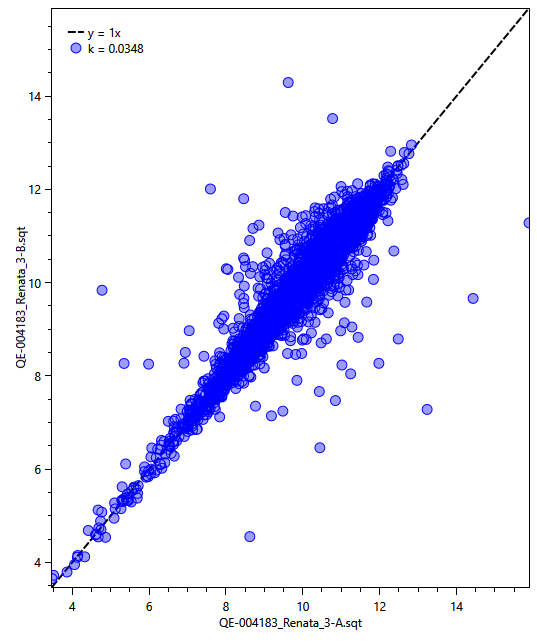

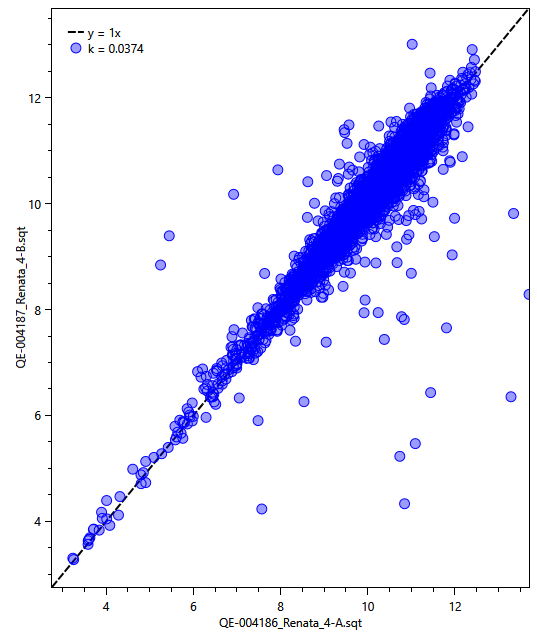

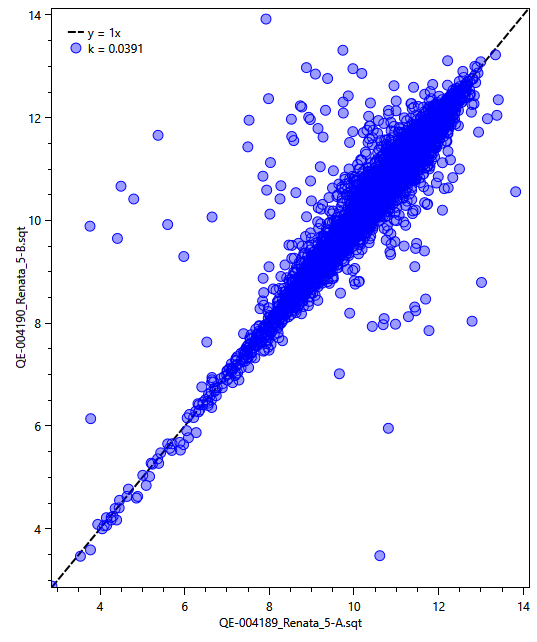

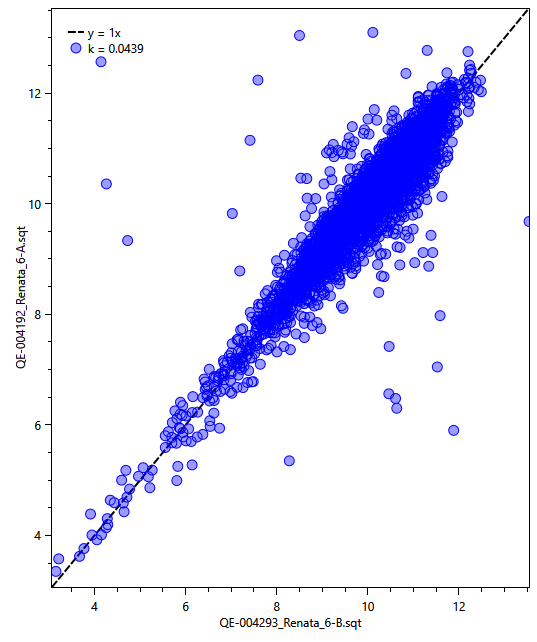

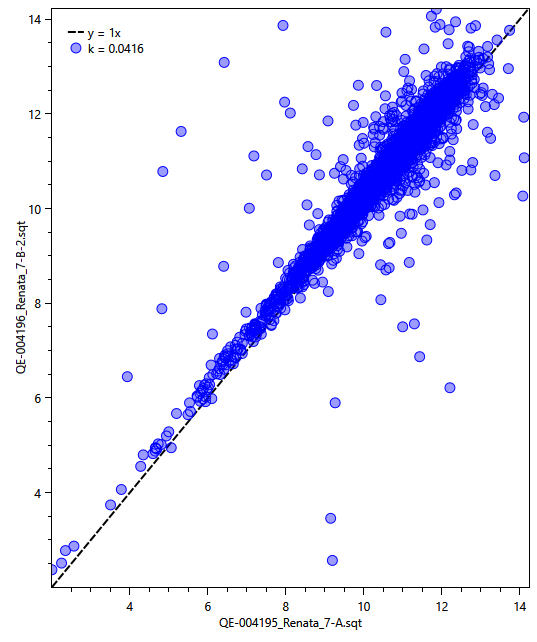

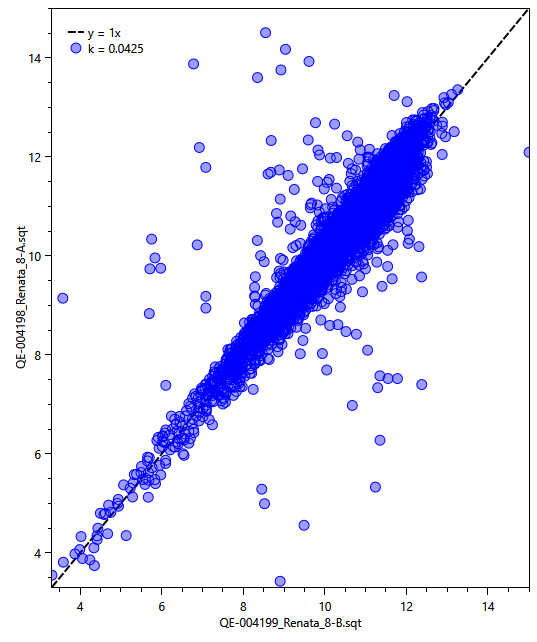

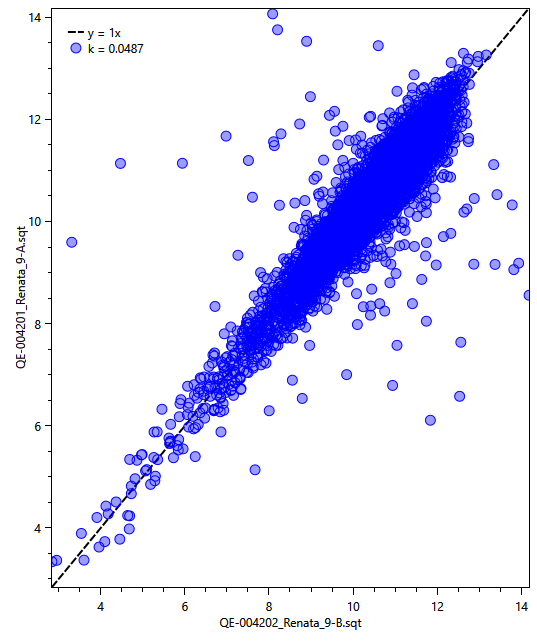

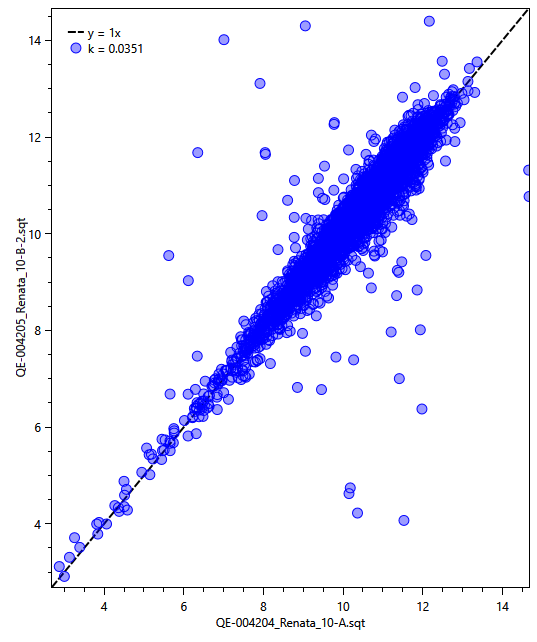

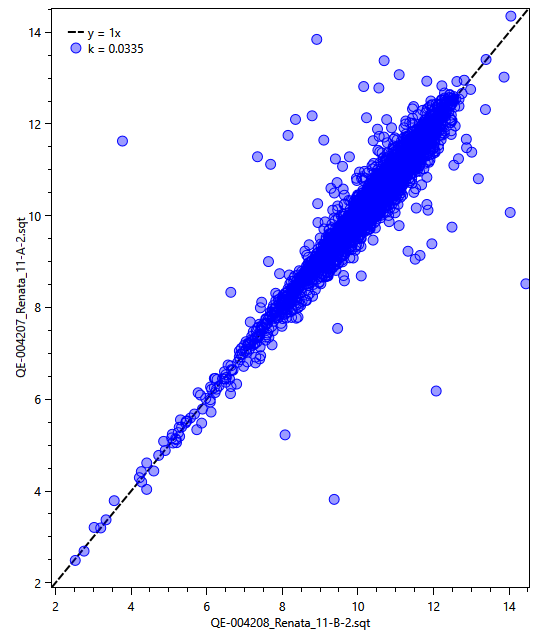

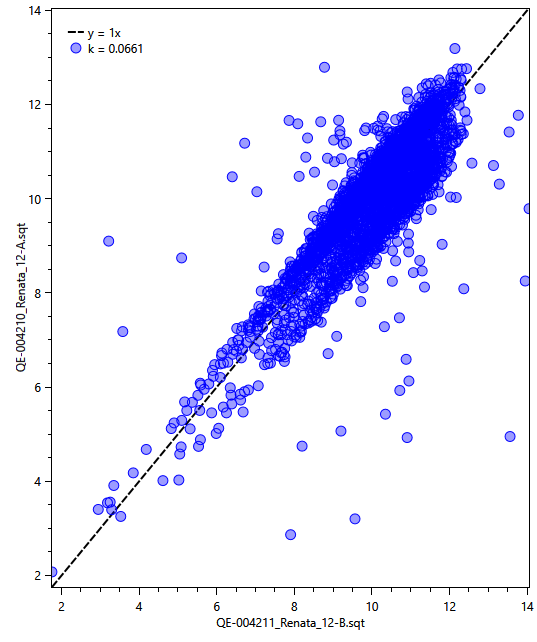

Supplement: Supplementary file 2 — Supplemenatry information2. [file 41598_2020_67113_MOESM2_ESM.docx]
